# Trends and predictors of inequality in childhood stunting in Nepal from 1996 to 2016

**DOI:** 10.1186/s12939-019-0944-z

**Published:** 2019-03-05

**Authors:** Mirak Raj Angdembe, Bishnu Prasad Dulal, Kreepa Bhattarai, Sumit Karn

**Affiliations:** 1 HERD International, Thapathali-11, Kathmandu, Nepal; 2 Social Development and Promotion Centre, Sanepa, Lalitpur, Nepal; 3 South Asian Infant Feeding Research Network – Nepal, Kathmandu, Nepal

**Keywords:** Concentration index, Decomposition, Predictors, Nepal, Nepal demographic and health survey, Socioeconomic inequalities, Stunting

## Abstract

**Background:**

Although decreasing in trend, one-in-three children remain stunted in Nepal and its distribution is unequal among different socioeconomic and geographical subgroups. Thus, it is crucial to assess inequalities in stunting for designing equity focused interventions that target vulnerable groups with higher burden of stunting. This study measures trends and predictors of socioeconomic inequalities in childhood stunting in Nepal.

**Methods:**

Data from five rounds (1996–2016) of Nepal Demographic and Health Survey, nationally representative cross-sectional surveys, were used. Levels and trends of absolute and relative disparity in stunting between the poorest and the richest wealth quintiles, and among all quintiles were assessed by calculating absolute and relative difference, concentration curve and index. Average marginal effects of predictors on stunting were calculated using probit regression. The concentration index was subsequently decomposed into contributing factors.

**Results:**

Even though stunting consistently declined in all wealth quintiles between 1996 and 2016, reduction was relatively higher among the richer quintiles compared to poorer ones. The absolute difference between the poorest and the richest quintile increased from 24.7 in 1996 (64.5% in poorest – 39.8% in richest) to 32.7 percentage points in 2016 (49.2–16.5%). The relative disparity also increased; the ratio of stunting in the poorest to the richest quintile was 1.6 in 1996 and 3.0 in 2016. The concentration index increased (in absolute value) from − 0.078 in 1996 to − 0.147 in 2016 indicating that stunting was disproportionately concentrated in poorer households and socioeconomic inequalities worsened from 1996 to 2016. Decomposition analysis revealed that in 1996, wealth (61%), caste/ethnicity (12%), mother’s education (12%) and birth order (9%) were the major contributors to observed socioeconomic inequalities in stunting; while in 2016, wealth (72%), mother’s BMI (12%) and birth order (9%) were the major contributors.

**Conclusions:**

Despite remarkable improvements in average stunting over the last two decades, substantial socioeconomic inequalities in stunting exists and is determined not only by immediate factors but also by underlying and contextual factors which emphasize the need for coherent actions across different sectors. In addition to reducing inequalities in wealth, nutrition programming should be focused on most disadvantaged subgroups which are prone to both stunting and relative poverty.

## Introduction

Under-nutrition is still a major barrier for child growth and development in developing countries. Low height-for-age or stunting is an important indicator for assessing undernutrition among children. It represents the devastated result of poor nutrition over a long period, in children under-five years [1]. Stunting can happen in the first 1000 days of child’s life after conception and is worsened by recurrent and chronic illnesses [2, 3]. Coupled with micronutrient deficiencies, affected children may suffer from irreversible brain damage, impeding their complete developmental potential. Even survivors are more likely to lead diminished lives, have compromised cognitive abilities, reduced school performance, lowered economic productivity and are at a greater risk of nutrition-related chronic diseases later in life [2, 4].

Globally, 150 million children under-five were stunted in 2017, of which, more than half (55%) were Asian [5]. Although undernutrition is decreasing globally, stunting is declining relatively at a slower pace, threatening the achievement of international commitments [6]. At 36% in 2016, stunting is still unacceptability high in Nepal with a slow rate of decline (57% in 1996 and 2001, 49% in 2006, 41% in 2011) [7–11]. Masked by national average, stunting and severe stunting in some parts of the country are even higher and wide variation between different socioeconomic and geographical subgroups is worrisome. In 2016, stunting was higher in the mountain region^1^ (47%) where access to health care and other services is particularly poor, Karnali province^2^ (55%), children born to mothers with no education (46%) and children belonging to households in the poorest wealth quintile (49%) [10].

Childhood stunting is considered to be the best overall indicator of children’s well-being that reflects social inequalities [12]. It is associated with a multitude of demographic, socioeconomic and nutritional factors such as child’s age, gender, dietary intake, household economic status, mother’s education, mother’s age and household food insecurity [2, 13]. Nevertheless, food insecurity, mother’s education and household income were considered as the most important predictors [2, 14–16]. Whereas the major contributors to socioeconomic disparities in stunting and their changes over time were household economic status and sanitation, parental education, utilization of health services (antenatal care, delivery at health facility), maternal short stature, child’s age, birth order, duration of breastfeeding and ethnicity [17–19].

The Government of Nepal is committed to achieving the Sustainable Development Goals (SDG) 2030 principled on ‘leaving no one behind’. These goals necessitate disaggregation of data by multiple dimensions including wealth [20]. With equity and access as one of the strategic pillars; inclusion and disaggregated data needs are recurring motifs in Nepal’s current health sector strategy 2015–2020 [21]. To provide momentum towards universal health coverage as envisioned by the sectoral strategy, an enriched understanding of who and where the disadvantaged and vulnerable children are, is thus important. To this end, efforts have been underway. Nepal joined the ‘scaling up nutrition’ movement in 2011 to strengthen political commitment and accountability for ending malnutrition. Efforts have also been seen in the form of nutrition specific and sensitive interventions through different sectors under the common framework of Multi-Sector Nutrition Plan [22]. However, these are still relatively recent and may not be widespread enough to reach the most vulnerable and impoverished population [15].

Given that one-in-three children remain stunted and its distribution is unequal among different population subgroups, understanding inequalities in stunting is crucial for designing equity focused interventions. In this context, this study aims to assess the levels and trends in childhood stunting by wealth quintile (a measure of household economic status) in Nepal; use absolute and relative measures including concentration index to capture inequality across all quintiles; and then ‘decompose’ these inequalities by quantifying the contributions attributable to each predictors and examine their changes over time. To our knowledge this is the first study to investigate drivers of disparities in stunting and their trends in Nepal.

## Methods

### Data

We used data from five rounds of Nepal Demographic and Health Surveys (NDHS) conducted in Nepal in 1996 (originally called Nepal Family Health Survey, NFHS), 2001, 2006, 2011 and 2016. The datasets were downloaded with permission from the DHS program. NDHS are nationally representative cross-sectional household surveys that provide data for a wide range of indicators in the areas of population, health and nutrition. They use two-stage or three-stage, systematic cluster random sampling design. Sample size and response rates are presented in Table 1. The NDHS reports can be referred to for further detail [7–11].

**Table 1:** Number of households, response rate, number of children and time of field work by survey year

	Survey year				
	1996	2001	2006	2011	2016
Total households (N)	8082	8602	8707	10,826	11,490
Response rate (%)	99.6	99.6	99.6	99.4	98.5
Total children^a^ (N)	3734	6367	5417	2430	2446
Total children^b^ (weighted N)	3703	6442	5258	2485	2421
Total children^c^ (weighted N)	2967	NA	NA	NA	1588
Time of field work	Jan-Jun 1996	Jan-Jun 2001	Feb-Aug 2006	Jan-Jun 2011	Jun 2016 – Jan 2017

^a^In 1996 anthropometric data were collected for under-three years old children; in 2001, 2006, 2011 and 2016 anthropometric data were collected for under-five years old children

^b^Sample size used for calculating the quintile specific trends, concentration curves, levels and trends in concentration indices and absolute and relative differences

^c^Sample size used for decomposition analysis to explain between-year changes in inequalities comparing 1996 and 2016

NA: Not applicable

The 1996 and 2001 surveys did not calculate the wealth index factor scores in original datasets. These are available as separate files. Similarly, the updated anthropometric z-scores based on 2006 World Health Organization’s (WHO) child growth standards [23] are also available as separate files for these surveys.. Data from these separate files were thus merged with the datasets of 1996 and 2001.

The 1996 survey collected anthropometric data for all children under-three years of age born in the three years before the survey to women interviewed. We used children’s recode dataset for all calculations of 1996. Other surveys collected anthropometric measurements for under-five years’ de facto children – those who stayed in the household the night before the interview. So except for 1996 survey, we used household member recode datasets which contain information for all children under-five in the household for calculating the quintile specific trends, concentration curves, levels and trends in concentration indices and absolute and relative differences (Weighted N: 1996–3703; 2001–6442; 2006–5258; 2011–2485; 2016–2421). For decomposition analysis of 1996 and 2016, we used children recode dataset for characteristics of children and their mother (Weighted N: 1996–2967; 2016–1588).

### Outcome variable

Compared to underweight and wasting, both of which reflect recent nutritional distress, stunting is a result of chronic nutritional deprivation [24]. Although all three indicators are equally important to measure nutritional imbalance resulting in undernutrition, we analysed stunting among children as outcome to better measure inequalities in long term nutritional progress in Nepal. Stunting was measured using height-for-age z-scores. The WHO child growth standard [23] expresses a child’s height in standard deviation units (z-scores) above or below the median height of healthy children in the same age group or in a reference group. Using this standard, we classified children whose height-for-age z-score was below minus two standard deviations (<− 2 SD) from the median of the reference population as short for their age (stunted) or chronically malnourished.

### Conceptual framework

The WHO’s conceptual framework on childhood stunting [25] explains the context, causes and consequences of stunting and underpins our analysis. The process of stunting begins even before birth. Maternal factors such as short maternal stature, intrauterine growth retardation, short birth spacing and poor nutrition during pre-conception, pregnancy and lactation contributes to stunted growth and development of the child. After birth, breastfeeding practices become important and household factors such as inadequate sanitation affects the risk of infection and morbidities that interfere with growth. Wider contextual factors, among many others, include wealth, education, socio-cultural factors such as caste/ethnicity, and access to health care. Together with wealth and education, caste/ethnicity determines the socioeconomic position of populations placing certain groups at an advantage in terms of access and use of resources while marginalizing others.

Differences in exposure and vulnerability to poor health outcomes are linked to people’s respective social status [26]. In Nepal, significant disparities in access to health care among people of different caste/ethnic groups exist [27]. Food consumption patterns [28] including complementary feeding practices [29] also vary between different sociocultural groups affecting their nutritional status. In health care access, antenatal care (ANC) visits and delivery in health facility are usually the first points of contact with the health system for most pregnant women. These are critical windows of opportunity for health programs to provide evidence-based interventions likely to prevent stunting in utero and later.

### Predictors of socioeconomic inequalities

We used the conceptual frame work and previously published literatures [13, 19, 30–32] that have shown strong association between either stunting or height-for-age z-scores and socioeconomic, maternal, child and Water, Sanitation and Hygiene (WASH) factors to guide the selection of our predictor variables. Our selections were also restricted by what was available in the 1996 NDHS dataset.

In *socioeconomic factors*, first we included household wealth index. Income and wealth enables access to better quality foods, health care and other factors that can raise the nutritional status of children [33]. The NDHS calculates wealth index (also called the asset index) composed of a set of variables asked in household questionnaires that describe household assets and utilities. It is a composite measure of a household’s cumulative living standard and is used as a proxy for household welfare. Households are given scores based on the number and kinds of consumer goods they own, ranging from a television to a bicycle or car, and housing characteristics such as source of drinking water, toilet facilities, and flooring materials [10]. Constructed using principal component analysis, scores for the first principal component gives the index and individual households are placed on a continuous scale of relative wealth. The quintiles are then constructed with each quintile containing 20% of the population.

Second we included caste/ethnicity. The caste variable recoded^3^ by NDHS was adapted for analysis into following groups: *Brahmin/Chhetri*, *Dalit*, *Janajati*, Muslim, *Newar*, other *terai* caste and others. The category ‘others’ was later omitted during model fitting to minimize errors. *Maternal and parental factors* included education level of mother and her husband/partner, height (cm), body mass index (BMI), ANC visits and delivery in a health facility. *Child characteristics* consisted of age (months), birth order, status of breastfeeding and perceived size of baby at birth. *WASH factors* comprised availability of toilet facilities in household. We used these predictor variables in decomposition analysis to explain between-year changes in inequalities comparing 1996 and 2016.

### Measurement of socioeconomic inequalities

We started with comparison of stunting across different wealth quintiles. We then used both absolute and relative measures of inequality for comprehensive assessment of household economic disparities in stunting, as using either one only can lead to dissimilar inferences about the magnitude and changes [34]. We calculated one absolute (the difference between stunting in the poorest/first and richest/fifth quintiles) and two relative indicators of inequality (the ratio of stunting in the poorest quintile to the richest quintile and the concentration curve/index).

The concentration curve and index captures inequality across all wealth quintiles. The concentration curve plots the cumulative proportion of stunting against the cumulative proportion of children, ranked by wealth index, beginning with the poorest, and ending with the richest (x-axis). We constructed concentration curves for 1996 and 2016 to illustrate changes in inequality in stunting between these survey periods and also applied statistical test of dominance [35] between the concentration curves to assess whether differences between curves are significant.

To quantify the degree of socioeconomic inequality in stunting we calculated the concentration index which is defined as twice the area between the stunting concentration curve and the line of equality (the diagonal or 45-degree line; see Fig. 2) [35]. The index is expressed in a scale ranging from − 1 to 1; a value of zero represents perfect equality, whereas a value of 1 to − 1 indicates that only the richest or the poorest household bear the burden of stunting. It is negative (positive) when the curve lies above (below) the line of equality, indicating a higher relative burden of stunting among the poor (rich). When we multiply the value of concentration index by 75 we get an estimation of the percentage of stunting to be redistributed from the richer half to the poorer half, in order to reach a distribution of perfect equality and to obtain an index value of zero [36]. In our analysis, since the outcome variable is dichotomous, the bounds of the concentration index are not − 1 and 1 but depend on the mean of the variable. To ensure robustness, we further normalized the standard index estimates by dividing through by 1 minus the mean [37] and presented the results.

The concentration index can be written in terms of the covariance between the outcome variable (stunting) and the fractional rank in the socioeconomic distribution (wealth index) as follows:

1 $$ C=\frac{2}{\mu } CO{V}_w\left({y}_i,{R}_i\right) $$

Here, *y*_*i*_ refers to the outcome of the i^th^ individual, *R*_*i*_ is the fractional rank of the i^th^ ranked individual in the socioeconomic distribution, while *μ* is the weighted mean of *y*, and *COV*_*w*_ denotes the weighted covariance.

### Decomposition of inequalities

To identify the contribution of each of the predictors to the observed socioeconomic inequality in stunting we decomposed the concentration index of stunting to reflect the proportional contributions of predictors, together with an unexplained residual component (ε). For any linear additive regression model explaining outcome (*y*), with a set of predictors (*k*), their regression coefficients (*β*_*k*_), the intercept (*α*), the relative contributions of *X*_*k*_ predictors and error term (*ε*) [35, 38] such as

2 $$ y=\alpha +{\sum}_k{\beta}_k{X}_k+\varepsilon $$

the concentration index for y (i.e. C) can be written as follows:

3 $$ C={\sum}_k\left(\frac{\beta_k{\overline{X}}_k}{\mu}\right){C}_k+\frac{GC_{\varepsilon }}{\mu } $$

Equation (3) shows that the overall inequality in outcome has two components, a deterministic or ‘explained’ component and an ‘unexplained’ component; one which cannot be explained by systematic variation in the predictors across wealth groups. In the deterministic component *β*_*k*_ is the coefficient from a regression of outcome on predictor *k*, ${\overline{X}}_k$ is the mean of predictor *X*_*k*_, *μ* is the mean of *y* and *C*_*k*_ is the concentration index for predictor *X*_*k*_ (defined analogously to C). In the unexplained component, *GC*_*ε*_ is the generalized concentration index for the error term (*ε*). Using the explained component, we can calculate the contribution of each predictor to inequality by multiplying the outcome elasticity (impact each predictor has on the outcome) with respect to that predictor and its concentration index (degree of unequal distribution) i.e. $\left(\frac{\beta_k{\overline{X}}_k}{\mu}\right){C}_k$.

Even if the contribution of a predictor is large, it will not have a large contribution to inequality, if it is equally distributed between the rich and the poor. Predictors that are more concentrated among the poor and associated with a higher probability of stunting, or those that are more prevalent among the rich and associated with a lower probability of stunting would lead to inequality. Thus, both the impact of the predictor on the outcome, as well as its distribution by economic status (given by concentration index) determines the contribution of predictors to total inequality. Next, the percentage contribution of each predictor can be estimated by dividing its absolute contribution by the concentration index of the outcome i.e. $\left(\frac{\beta_k{\overline{X}}_k}{\mu}\right){C}_k/C$.

The decomposition method was first introduced for use with linear prediction models [38]. However, in our study the outcome variable is binary and thus requires non-linear statistical techniques. Of the two popular choices – the logit and the probit model that yield probabilities in the range (0,1) and are fitted by maximum likelihood; we used the probit model which has also been used in another study [19]. Because the normal and logistic distributions are similar, the choice between a probit or a logit specification is not important in most cases [35].

4 $$ {h}_i={\alpha}^m+{\sum}_k{\beta}_k^m{X}_{ki}+{u}_i $$

In a probit model, using marginal or partial effects (*dh/dx*), which gives the change in predicted probability associated with unit change in predictor variable, allows for dealing with discrete changes from 0 to 1. Thus, this approximation of non-linear relationship using marginal effects restores the mechanism of the decomposition framework in eqs. (2) through (4) [39]. Equation (4) gives the linear approximation of the non-linear estimations, where u_i_ indicates the error generated by the linear approximation used to obtain the marginal effects. This method has been used previously in analysis of health sector inequalities [40, 41]. We calculated average marginal effects from predictions of probit model and a two-tailed *P* value < 0.05 was considered statistically significant. All the estimates take sampling weights into consideration. We used Stata 15.1® for statistical analyses.

## Results

### Socioeconomic inequalities

**Fig. 1:**
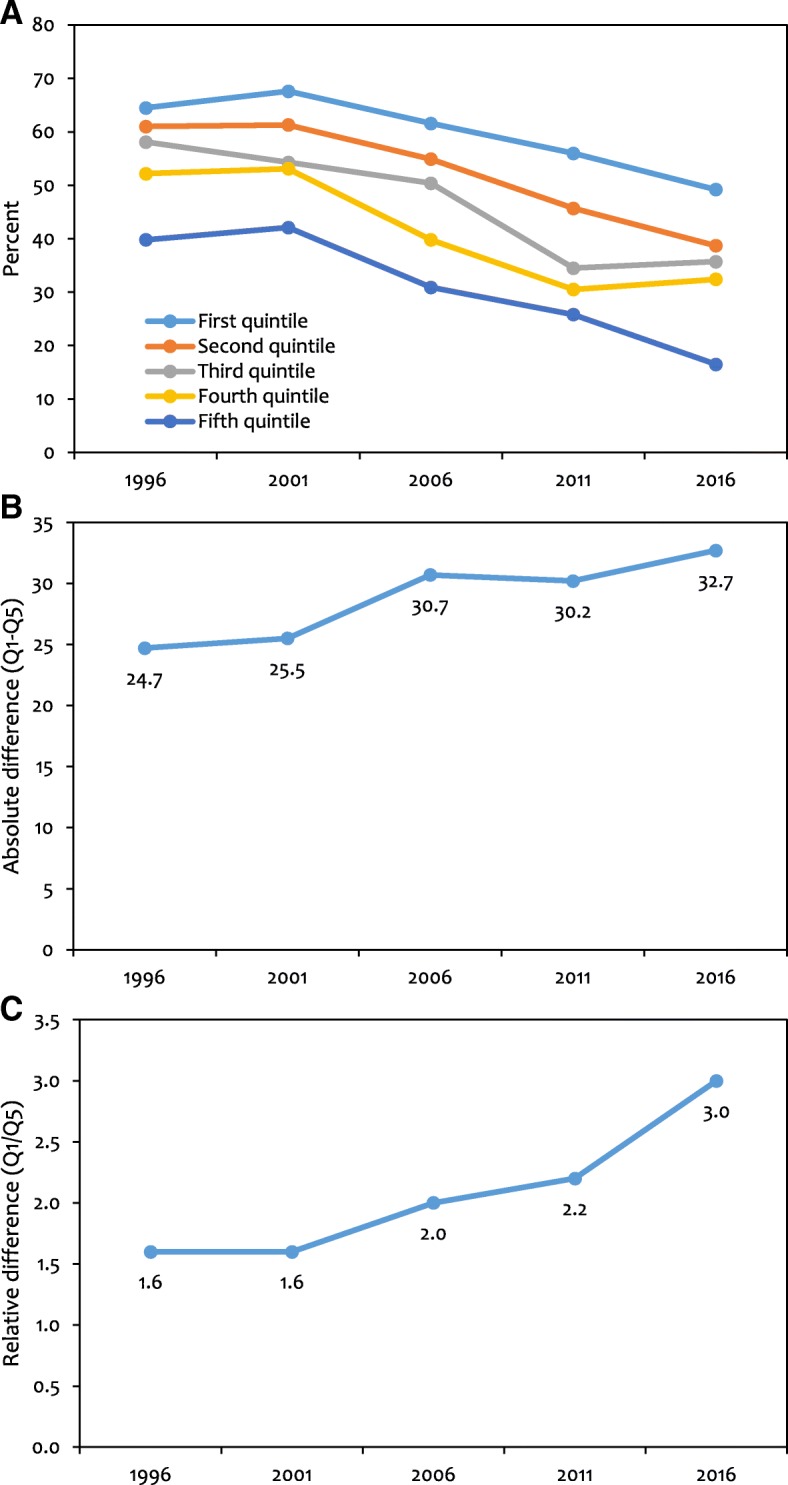
Quintile-specific trends in stunting from 1996 to 2016. (**a**) Stunting prevalence (**b**) Absolute difference (Q1-Q5) (**c**) Relative difference (Q1/Q5). The first quintile (Q1) is the 20% poorest quintile and the fifth quintile (Q5) is the 20% richest. (Weighted N: - 1996: 3703; 2001: 6442; 2006: 5258; 2011: 2485; 2016: 2421)

Stunting has consistently declined in all wealth quintiles between 1996 and 2016. However, the reduction was relatively higher among the richer quintiles compared to the poorer ones. In 1996, stunting was 64.5% (95% CI: 61.4 to 67.7) in the poorest (first) quintile and 39.8% (35.5 to 44.0) in the richest (fifth) quintile (Table 2, Fig. 1). By 2016, stunting dropped in Nepal to 49.2% (44.8 to 53.6) in the poorest quintile, 16.5% (11.7 to 21.3) in the richest quintile, and in between these outcomes in the middle quintiles. The average decline per year during this period was 0.8 percentage points in the poorest quintile compared to 1.2 percentage points in the richest quintile which was the highest rate of reduction among all wealth groups.

**Table 2:** Trends and estimates for quintile-specific stunting in 1996, 2001, 2006, 2011 and 2016

	Stunting (95% confidence intervals)					Absolute decline in stunting (percent points)		Percentage decline in stunting (%)	
						Average decline per year	Total decline	Average decline per year	Total decline
	1996	2001	2006	2011	2016	1996–2016	1996–2016	1996–2016	1996–2016
First quintile (poorest)	64.5 (61.4 to 67.7)	67.6 (65.2 to 70.1)	61.6 (58.7 to 64.5)	56 (51.7 to 60.2)	49.2 (44.8 to 53.6)	0.8	15.3	1.3	23.7
Second quintile	61 (57.3 to 64.7)	61.3 (58.4 to 64.1)	54.9 (51.3 to 58.4)	45.7 (40.4 to 51.0)	38.7 (34.0 to 43.3)	1.1	22.3	2.2	36.6
Third quintile	58.1 (54.3 to 62.0)	54.3 (51.3 to 57.3)	50.4 (46.6 to 54.1)	34.5 (29.4 to 39.7)	35.7 (31.1 to 40.3)	1.1	22.4	2.4	38.6
Fourth quintile	52.2 (48.4 to 55.9)	53.1 (50.0 to 56.1)	39.8 (36.1 to 43.4)	30.5 (24.7 to 36.3)	32.4 (27.5 to 37.4)	1.0	19.8	2.4	37.9
Fifth quintile (richest)	39.8 (35.5 to 44.0)	42.1 (38.9 to 45.4)	30.9 (26.9 to 35.1)	25.8 (20.1 to 31.5)	16.5 (11.7 to 21.3)	1.2	23.3	4.3	58.5
Total stunting	56.6 (54.9 to 58.2)	57.2 (55.9 to 58.5)	49.3 (47.7 to 50.9)	40.5 (38.1 to 42.9)	35.8 (33.7 to 38.0)	1.0	20.8	2.3	36.7
Ratio of first to fifth quintile stunting	1.6	1.6	2.0	2.2	3.0				
Difference in first and fifth quintile stunting	24.7	25.5	30.7	30.2	32.7				
Concentration index (95% confidence intervals)	−0.078 (− 0.094 to − 0.061)	− 0.083(− 0.095 to − 0.070)	− 0.125(− 0.143 to − 0.108)	− 0.164 (− 0.194 to − 0.134)	− 0.147(− 0.179 to − 0.114)				
Standard error of concentration index	0.008	0.006	0.009	0.015	0.017				
Normalized concentration index^a^	− 0.178	− 0.193	− 0.247	− 0.276	− 0.228				
Weighted N	3703	6442	5258	2485	2421				

^a^Normalization of concentration index involves dividing the concentration index by (1 – proportion stunted)

The largest absolute decline in stunting prevalence between 1996 and 2016 occurred in the richest quintile with 23.3 percentage points. The corresponding percentage decline between 1996 and 2016 was 58.5% which took place at the rate of 4.3% per year (Table 2). As a result of the greater absolute decline in stunting in the richest quintile than in the poorest quintile, the difference between these quintiles increased from 24.7 in 1996 to 32.7 in 2016 (Table 2, Fig. 1). The relative disparity between the poorest and the richest groups has also increased; the ratio of stunting in the first quintile to fifth quintile was 1.6 in 1996 and 3.0 in 2016.

**Fig. 2:**
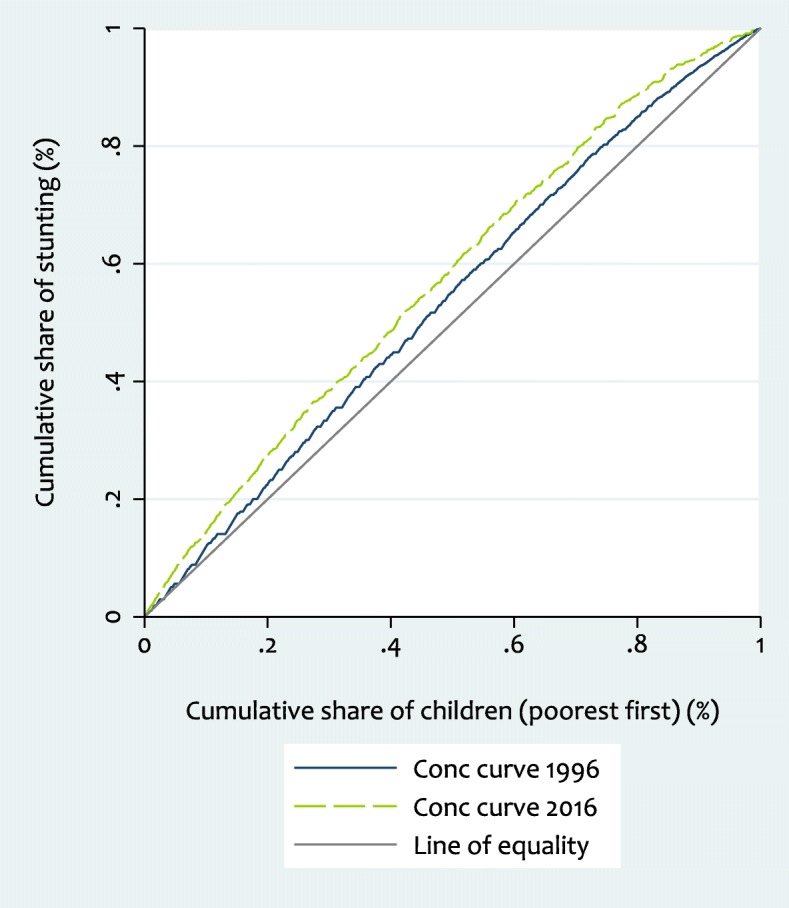
Concentration curve for stunting, 1996 and 2016 (Weighted N: - 1996: 3703; 2016: 2421)

Figure 2 presents concentration curves for stunting in 1996 and 2016. In 1996, the concentration curve was consistently above the line of equality, which means stunting was disproportionately concentrated in poorer households. In 2016, the curve has shifted even further away from the line of equality, showing increase in degree of inequality over the years. In order to obtain zero inequality, it is necessary to redistribute 5.9% stunting (not stunted) in 1996 and 11% in 2016 from the richer to the poorer half of the population. Here, we can reject the null of non-dominance at the five percentage level of significance using the less strict option within the test described in O’Donnell et al. [35]. This means the 2016 concentration curve dominates (lies above) that of 1996 but the two curves overlap toward the bottom of the wealth distribution.

**Fig. 3:**
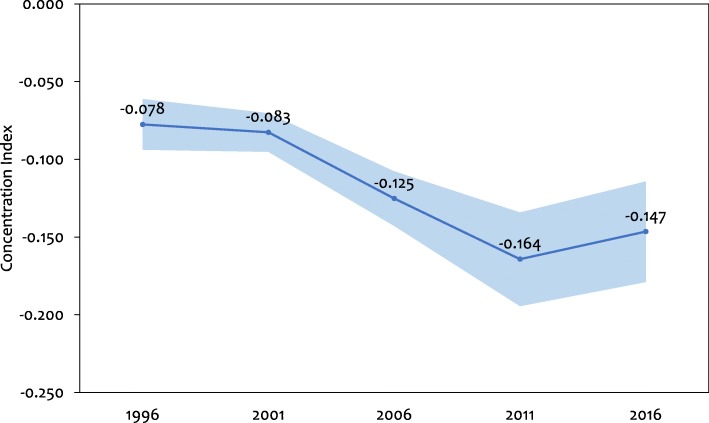
Trend in concentration index of stunting with 95% confidence intervals. Curve illustrates point estimates. Shaded areas are 95% confidence intervals. (Weighted N: - 1996: 3703; 2001: 6442; 2006: 5258; 2011: 2485; 2016: 2421)

In addition, the concentration index increased (in absolute value) from − 0.078 (95% CI: -0.094 to − 0.061) in 1996 to − 0.164 (− 0.194 to − 0.134) in 2011 (Table 2, Fig. 3). It decreased slightly from 2011 to 2016 (− 0.147, 95% CI: -0.179 to − 0.114). The negative values indicate that stunting was disproportionately concentrated in poorer households. We find that socioeconomic inequalities in stunting, as measured by concentration index, worsened from 1996 to 2011, however improved somewhat in 2016. The ‘normalized’ concentration indices show even stronger evidence of the burden of stunting being more concentrated among the poor (Table 2).

### Marginal effects of predictors

In mothers having a higher BMI the chances of stunting were significantly reduced in 2016 (Normal, *P* = 0.042 and Overweight/Obese, *P* = 0.003). Results were similar in 1996, but statistically non-significant (Table 3). Mothers with ‘secondary and above’ education had significantly lower probability (*P* = 0.020) of stunting in 1996. Although results were similar in 2016 i.e. higher education decreasing the chances of stunting, it was not statistically significant. Mothers with short height (< 145 cm) were significantly more likely (*P* < 0.001) to have stunted children both in 1996 and 2016 with higher effect in 2016.

**Table 3:** Decomposition of the concentration index for stunting (1996 & 2016)

Variables	Marginal effect				Weighted mean (1996: Mean_stunted_ = 0.553; 2016: Mean_stunted_ = 0.337)		Concentration Index (C) (1996: C_stunted_ = − 0.082;2016: C_stunted_ = − 0.138)		Absolute contribution		Percentage contribution (%)		Change
	1996	*P* value	2016	*P* value	1996	2016	1996	2016	1996	2016	1996	2016	
Mother’s education													
Primary	−0.030	0.331	− 0.048	0.173	0.113	0.186	0.157	−0.179	− 0.0010	0.0048	1.2	−3.7	−4.9
Secondary and above	−0.085*	**0.020**	−0.023	0.558	0.099	0.480	0.575	0.191	−0.0087	−0.0062	11.0	4.7	−6.3
Sub-total									**−0.0097**	**− 0.0014**	**12.2**	**1.1**	**− 11.1**
Husband/Partner’s education													
Primary	−0.035	0.143	0.015	0.725	0.244	0.231	−0.070	−0.257	0.0011	−0.0027	−1.4	2.1	3.5
Secondary and above	−0.035	0.172	0.039	0.418	0.389	0.624	0.243	0.153	−0.0059	0.0110	7.4	0.8	−6.6
Sub-total									**−0.0048**	**0.0083**	**6.1**	**2.9**	**−3.2**
Maternal short stature (< 145 cm)	0.137*	**< 0.001**	0.253*	**< 0.001**	0.150	0.114	−0.007	− 0.089	− 0.0002	− 0.0077	0.3	5.9	5.6
Mother’s BMI													
Normal (18.5 to < 25)	−0.018	0.446	−0.069*	**0.042**	0.711	0.634	−0.006	−0.046	0.0001	0.0060	−0.2	−4.6	−4.4
Overweight/Obese (≥25)	−0.065	0.344	−0.148*	**0.003**	0.018	0.176	0.350	0.285	−0.0007	−0.0221	0.9	16.9	16.0
Sub-total									**−0.0006**	**−0.0161**	**0.7**	**12.3**	**11.6**
Antenatal care visits	−0.002	0.930	−0.040	0.532	0.438	0.943	0.172	0.023	−0.0002	−0.0025	0.3	1.9	1.6
Delivery in health facility	−0.046	0.157	−0.007	0.806	0.082	0.573	0.573	0.179	−0.0039	−0.0021	4.8	1.6	−3.2
Children aged 25–59 months	0.172*	**< 0.001**	0.109*	**< 0.001**	0.272	0.502	0.005	0.019	0.0004	0.0031	−0.5	−2.4	−1.9
Birth order													
2nd - 4th	0.076*	**0.001**	0.070*	**0.021**	0.525	0.574	0.006	−0.009	0.0005	−0.0011	− 0.6	0.8	1.4
5 or more	0.109*	**< 0.001**	0.130*	**0.007**	0.254	0.093	−0.154	− 0.281	− 0.0077	−0.0101	9.7	7.7	−2.0
Sub-total									**−0.0072**	**−0.0112**	**9.1**	**8.6**	**−0.5**
Breast fed children	−0.189	0.511	−0.186	0.150	0.998	0.993	−0.001	−0.0004	0.0002	0.0002	−0.2	−0.2	0.0
Perceived size of baby at birth													
Average	0.052*	**0.014**	0.032	0.311	0.431	0.672	0.021	0.020	0.0008	0.0013	−1.1	−1.0	0.1
Small	0.156*	**< 0.001**	0.084*	**0.039**	0.259	0.164	−0.080	−0.113	−0.0058	−0.0047	7.3	3.6	−3.7
Sub-total									**−0.0050**	**−0.0034**	**6.2**	**2.6**	**−3.6**
Caste/Ethnicity													
*Janajati*	−0.144*	**< 0.001**	−0.046	0.253	0.308	0.301	−0.013	−0.064	0.0010	0.0026	−1.3	−2.0	−0.7
Other *terai* caste	−0.071	0.068	−0.007	0.880	0.124	0.174	−0.020	0.165	0.0003	−0.0006	−0.4	0.5	0.9
Muslim	−0.083	0.076	−0.0002	0.998	0.058	0.059	0.068	0.185	−0.0006	0.0000	0.7	0.0	−0.7
*Newar*	−0.199*	**< 0.001**	−0.046	0.665	0.050	0.035	0.490	0.151	−0.0089	−0.0007	11.1	0.6	−10.5
*Brahmin/Chhetri*	−0.103*	**0.002**	−0.015	0.714	0.296	0.286	0.029	−0.025	−0.0016	0.0003	2.0	−0.2	−2.2
Sub-total									**−0.0098**	**0.0016**	**12.1**	**−1.2**	**−13.3**
Wealth quintile													
Second	−0.040	0.110	−0.061	0.133	0.213	0.218	−0.291	−0.377	0.0044	0.0150	−5.6	−11.5	−5.9
Third	−0.040	0.137	−0.124*	**0.002**	0.194	0.215	0.116	0.057	−0.0016	−0.0045	2.0	3.5	1.5
Fourth	−0.101*	**< 0.001**	−0.098*	**0.015**	0.197	0.220	0.507	0.492	−0.0182	−0.0316	22.8	24.2	1.4
Fifth (Richest)	−0.145*	**0.001**	−0.200*	**< 0.001**	0.148	0.144	0.852	0.856	−0.0330	−0.0732	41.4	56.1	14.7
Sub-total									**−0.0484**	**−0.0943**	**60.7**	**72.3**	**11.6**
Open Defecation	−0.039	0.253	0.050	0.222	0.812	0.199	−0.165	−0.171	0.0095	−0.0050	−11.9	3.9	15.8
Total sum contribution									−0.0797	−0.1305			
Residual (Unexplained)									−0.0021	−0.0076			

Weighted N: - 1996: 2967; 2016: 1588. Average marginal effects and *P* values were calculated from predictions of probit model. The reference groups used were mothers with no education, husband/partner with no education, mothers who are not short, mothers who are underweight (BMI < 18.5), mothers who did not visit health facility for ANC, mothers who delivered in places others than a health facility, children aged 0–24 months, first born children, children who are not breastfed, perceived size of baby at birth large, caste/ethnicity *Dalit*, first wealth quintile (poorest), households with toilet facilities. The contributions of predictors to the observed socioeconomic inequality in stunting is calculated according to eq. (3) by using means, concentration indices of predictors and marginal effects. The total contribution is the sum of absolute contributions or the overall concentration index minus the residual. ^*^p < 0.05

Children aged 25–59 months had significantly higher chances (*P* < 0.001) of stunting and it did not change over the study period (Table 3). Likewise, in 1996 (*P* < 0.001) as well as 2016 (*P* = 0.007), higher birth order was significantly associated with increased probability of stunting; effects were similar and were largest for children born fifth or later. Small (perceived) size of baby at birth had significantly higher probability of stunting in 1996 (*P* < 0.001) as well as 2016 (*P* = 0.039). Those of *Janajati* (*P* < 0.001), *Newar* (*P* < 0.001) and *Brahmin/Chhetri* (*P* = 0.002) origin were significantly less likely to be stunted in 1996, the effect being largest in *Newar*. In contrast, such effects were not significant in 2016. The upper two wealth quintiles had significant negative associations with probability of stunting in 1996 (Fourth, *P* < 0.001 and Fifth, *P* = 0.001); additionally, in 2016, the middle/third quintile also showed significant negative association (*P* = 0.002). In both years, largest effects were seen in the richest quintile.

### Decomposition of inequalities

In Table 3 which presents decomposition analysis, the concentration index for stunting was − 0.082 in 1996 and − 0.138 in 2016 (Weighted N: 1996–2967; 2016–1588), indicating that stunting was concentrated amongst the poor, more in 2016 than in 1996. The absolute contribution of each predictor was obtained by multiplying its marginal effect by its mean and concentration index, then dividing by the mean of stunting (0.553 in 1996 and 0.337 in 2016). For example, the contribution of being in the richest wealth quintile in 1996 can be computed as: Marginal effect (− 0.145) *Mean (0.148) *Concentration Index (0.852) divided by weighted mean of stunting (0.553) = − 0.0330. Likewise, for 2016, (− 0.200) *(0.144) *(0.856)/ (0.337) = − 0.0732. Positive (negative) contributions of predictors indicate that the total inequality would, ceteris paribus, be lower (higher) if that predictor had no impact on stunting (instead of that reflected in marginal effects) or was equally distributed across the socioeconomic spectrum (instead of concentrated, as mirrored in the concentration indices of predictors) [41]. The corresponding percentage contributions can be calculated by dividing the contribution of each predictor by the total (sum contribution) explained portion of the concentration index (− 0.0797 in 1996 and − 0.1305 in 2016), which is = − 0.0330/− 0.0797 = 41.4% in 1996 and − 0.0732/− 0.1305 = 56.1% in 2016. Therefore, being in the richest wealth quintile contributed to 41.4% of the inequalities in stunting in 1996, which is lower than the contribution of 56.1% in 2016.

Similarly, in Table 3, where overall stunting is concentrated amongst the poor (negative concentration index) we can interpret the contributions of individual predictors to the overall inequality as follows. Children born fifth or later (birth order) had an above average probability of stunting (positive marginal effect), were disproportionately concentrated in lower income groups (negative concentration index), and thus contributed − 0.0077 or 9.7% in 1996 and − 0.0101 or 7.7% in 2016 to the total observed inequality in stunting; a decrease in contribution of two percentage points. Since these contributions have the same sign as the overall concentration index, which indicates that stunting was concentrated amongst the poor, these indicate that children born fifth or later were a major pool of poor people with stunting. In perceived size of baby at birth, the 3.6% contribution of babies born small in 2016 was lower than the 7.3% contribution estimated in 1996; a decrease in contribution of 3.7 percentage points. In the same way we can compare contributions across categories of a predictor. For example, in 2016 the contribution of babies born 2^nd^ – 4^th^ in order was only 0.8%; lower than that contributed by those born 5^th^ or later (7.7%).

We can interpret a contribution with opposite sign to that on the overall concentration index in the following way. In 1996, children aged 25–59 months had a higher probability of stunting than children aged 0–24 months (the reference group) – a significant positive marginal effect of 0.172. However, since they were disproportionately concentrated in the higher income group (positive concentration index of 0.005), their contribution of 0.0004 was in the opposite direction to the overall inequality observed.

Analysing caste/ethnicity in 1996 we find that there is negative association between being a *Newar* and stunting (negative marginal effect); and with *Newar* being a rich group (positive concentration index) results in being of *Newar* origin contributing − 0.0089, or 11.1%, to the total inequality; which is higher than the contribution of just 0.6% in 2016; a decrease of 10.5 percentage points. In mother’s education, a negative association between those with secondary level and above education and stunting (negative marginal effect) and their concentration among the richer households (positive concentration index), has led to higher education level of mothers contributing − 0.0087, or 11.0%, to the total inequality. In 2016, this contribution decreased by 6.3 percentage points and was just 4.7%.

Maternal short stature was positively associated with stunting (positive marginal effect), and as those with short height were disproportionately poor (negative concentration index) they contributed − 0.0002, or 0.3%, in 1996 and − 0.0077, or 5.9%, in 2016; an increase in contribution of 5.6 percentage points to the total inequality. A higher mother’s BMI was negatively associated with stunting (negative marginal effect), and since those who were overweight/obese were disproportionately rich (positive concentration index) they contributed − 0.0007, or 0.9%, in 1996 and − 0.0221, or 16.9%, in 2016; an increase in contribution of 16 percentage points to the total inequality. In the same way we can interpret other decomposition results.

**Fig. 4:**
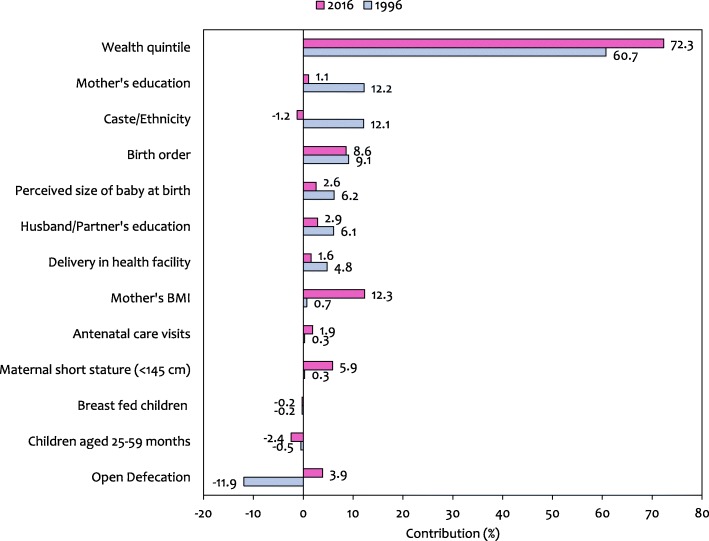
The percentage contribution of predictors to the total inequality in stunting in Nepal in 1996 compared to 2016. (Weighted N: - 1996: 2967; 2016: 1588)

We can also compare the overall contribution of a particular predictor by summing up contributions across its categories (reflected in sub-total, Table 3 and Fig. 4). In 1996, the major contributors to the observed socioeconomic inequalities in stunting were: wealth (60.7%), mother’s education (12.2%), caste/ethnicity (12.1%) and birth order (9.1%). ANC visits, maternal short stature and mother’s BMI played a less important role in terms of inequalities. In 2016, wealth (72.3%), mother’s BMI (12.3%) and birth order (8.6%) were the major contributors while mother’s education, delivery in health facility and ANC visits did not contribute considerably. Wealth was the biggest contributor to inequality in stunting and its contribution has increased by 11.6 percentage points between 1996 and 2016. The contribution of caste/ethnicity and mother’s education has decreased, while that of mother’s BMI has increased. The contribution of birth order to the total inequality in stunting has more or less remained constant.

## Discussion

From 1996 to 2016, Nepal’s political and economic climate was marred by an armed conflict, unstable governments, a massive earthquake with subsequent economic blockade by its neighbour, and transition to a new federal governance structure. In spite of such adversities, on an average, Nepal made remarkable ground in maternal and child nutrition indicators [30, 32]. However, with national averages we cannot identify those who are being left behind. Thus, using data from periodic surveys conducted during the 20-year time period, we analysed trends and predictors of inequalities in chronic malnutrition, stunting. Our analysis revealed widening disparity in stunting between the poorest and richest households both in absolute and relative terms. The better off have experienced larger and faster decline in stunting compared to the least affluent. Household economic status was the biggest contributor to inequality in stunting and its contribution has increased.

Although stunting has decreased in all wealth quintiles, the absolute gap between the poorest and richest households has increased, with consistently lower (higher) prevalence in richest (poorest) group. The absolute decline in stunting in the richest households was nearly 50% higher than those in the poorest households. Usually, as coverage of health interventions increase, the rich selectively benefit first, then only the poorest will lag behind all other groups [42, 43]. This was evident in our study where absolute disparities were smaller when stunting prevalence was high – possibly because most of the population, including the richest households, had inadequate access to nutrition interventions. But as stunting declined over time, largely driven by the faster decline among the richest households, stunting persisted in the poor with relatively lower rates of decline. Likewise, the relative difference between the poorest and richest stunting prevalence has also increased. By 2016 three times more children were stunted in the poorest quintile as compared with those in the richest quintile. This is expected when prevalence at the national level is decreasing. As stunting decline among the poor is outpaced by that in the rich, the absolute differences become higher which leads to higher ratios or relative inequalities.

The concentration curve and index analyses also revealed pro-rich improvements which have become more pronounced over time. Other studies from the region show similar patterns where stunting is declining on one hand while socioeconomic inequality is increasing on the other [18, 19, 44]. A lack of association between the rate of decline in stunting and improvement in equity was also reported in a multi-country analysis of survey data [45]. This unwanted trend in light of the government’s explicit commitment to enhance equity [21, 22, 46] questions the strategies and also the effectiveness of nutrition interventions. While government policies and programs are pro-poor and pro-inclusive, these results provide a strong case that stunting is distributed unequally across different socioeconomic subgroups in the population.

From decomposition analysis it is clear that most of the inequality was due to household economic status both in 1996 and 2016. In Nepal, improved access to healthcare, improvements in sanitation mainly use of toilets, improvements in (mother’s) education and particularly wealth accumulation have been identified as the key drivers of reduction in undernutrition [30, 32]. It is fair to say that nutrition sensitive interventions that address the underlying determinants of nutrition, including poverty and that draw on complementary sectors such as agriculture, health, social protection, early child development, education, and water and sanitation [47, 48] are more important for addressing inequities in stunting. Social safety nets that raise income among vulnerable groups and make them resilient to economic shocks are needed [48]. By redistributing income to the poorest and most vulnerable, they not only have an immediate impact on poverty and inequality [49] but also increase use of health and education services [50]. In this regard, there is much to learn from Bangladesh where pro-poor and women-focused investments in health and social development have contributed to equity gains in child survival [51]. One intervention that stands out is women’s microcredit that aimed to reduce poverty by providing poor families with access to small collateral-free loans. They targeted the ultra-poor women, encouraging economic and social empowerment and has had various positive effect on health outcomes [51]. Nevertheless, given the limited health system capacity, we need to first better understand the cost effectiveness and desirability of social safety net programs in Nepal, as in the past they have been found to have unintended negative consequences [52].

Similar studies in the region have noted mother’s education to be a prime contributor to inequalities in stunting [18, 19, 53]. Better education will not only contribute to higher household income but may also result in improved health knowledge, greater access to and use of health services, healthier feeding habits and enhanced decision making power within the household [54, 55]. In Nepal, more girls are getting education than ever before. The ratio of girls to boys in primary education increased from 0.56 in 1990 to 1.09 in 2015 [56]. This is a result of efforts to increase public expenditure in education. It started in the early 1990s and was provided further momentum in 2001 when the education for all national plan of action was adopted that brought gender equality and social inclusion to the forefront [30]. As a result, not only rich but poor women may also be getting educated, thus reducing the contribution of mother’s education to overall inequality.

With 125 caste/ethnic groups [57], Nepal’s population is diverse in caste/ethnicity. The contribution of caste/ethnicity to inequality in stunting has decreased, largely due to reduction in contribution of *Newar*. Stunting in *Newar* has become lesser pro-rich in 2016 compared to 1996, which means inequality has decreased. In general, *Newar* have a better health status compared to others. In 2016, they had the highest levels of institutional delivery, demand satisfied for family planning and children fully immunized; and lowest prevalence of anaemia and thinness (BMI < 18.5) among women. They also had the lowest rates of under-five and neonatal mortality rate and total fertility rate. In contrast, Muslim, other *Terai* caste and *Dalit* had relatively lower levels of service utilization and poorer health outcomes [58]. During the period of notable poverty reduction in Nepal between 1995 and 2010 when national poverty rates fell from 64 to 25% [59]; among all caste/ethnic groups, *Newar* consistently had lowest poverty rates, while *Dalit* stood on the bottommost end of the spectrum [60, 61].

The contribution of mother’s BMI to inequality has increased, owing to bigger contribution of overweight/obese mothers to inequality. Both in 1996 and 2016, stunting among children of overweight/obese mothers was disproportionately concentrated in richer households. In fact, a previous study has shown that compared to poorest households, the odds of being overweight and obese were higher among adult women belonging to the richest households [62]. However, compared to 1996, not only there was significant negative association between overweight/obese mothers with probability of stunting but also the proportion of mothers who were overweight/obese increased from just 1.8 to 17.6% in 2016. Other studies based on DHS data also show increasing prevalence of overweight and obesity among women of reproductive age [63, 64]. The rich poor differentials and growing prevalence of overweight/obesity has made mother’s BMI a major contributor to inequality. These are indications of changing lifestyle and highlights the need to adjust the existing strategies so that multiple forms of malnutrition can be addressed.

Similar to our results, a previous study reported significant association between higher birth order and stunting among children in Nepal [13]. Additionally, height-for-age z-scores, was also found to have significant negative association with higher fertility [65]. As opposed to 1996, the proportion of children born fifth or later has decreased in 2016, from 25.4 to 9.3% in our analysis; but this effect has been nullified by the increase in inequality – stunting concentration becoming even higher among the poor. In Nepal, even though women are having fewer children than before, the decline in total fertility rate in the poorer households is relatively slower compared to that in the richer households. In 2016, the total fertility rate was highest in the poorest wealth quintile at 3.2 compared to 1.6 in the richest quintile [10], which explains the negative shift of the concentration index in 2016 with regards to birth order. This is possibly the reason why contributions of birth order to inequality has remained similar over the years. A higher fertility not only has adverse consequences on mother’s health, but also affects the child’s birth weight and the mother’s ability to feed and care for her child [18], which are possible pathways to stunting. This highlights the need to further improve family planning practices among the poor.

Maternal short stature has intergenerational effects as measured by height of the mother on stunting [66]. A shorter height may lead to intrauterine growth retardation [67] and low birth weight [68], causing mortality and impaired child growth [2]. With higher positive effect and pro poor distribution, the contribution of maternal short stature to inequality has increased. Similarly, the concentration of small size of the baby at birth (perceived) has increased among the poor but due to its reduced positive effect on stunting as well as reduced prevalence, its overall contribution to inequality has decreased. A generation of poor women who were stunted as children may have given birth to children with similar impaired growth. Thus, it is crucial to formulate strategies that incorporate a life cycle approach to address nutrition issues during critical periods from conception to adulthood. Integration of nutrition counselling across maternal and child health and family planning interventions is also equally important. However, any new interventions and/or changes will need to be reflected at all levels of the government, especially at the local level, where services are delivered.

Improvements in availability of health care services have been identified to be among the key drivers of reduction in undernutrition in Nepal [30, 32, 65]. We examined the contributions of ANC visits and institutional deliveries which have reduced overtime and together accounted for only 3.5% of inequality in 2016. Utilization of these services has improved remarkably over the years. But what is even more noteworthy is the reduction in stunting inequality in these groups of mothers as indicated by the large shift of concentration index towards the null. Yet still the quality of ANC services is a concern that threatens these gains made. Only 17% of health facilities in Nepal offered high quality ANC services in 2015 [69]. Poor-quality care is now a bigger barrier than insufficient access in countries like Nepal where multi stakeholder commitment is critical for quality improvements [70].

While interpreting results, the following caveats will need to be considered. First, although decomposition of concentration index helps identify factors that potentially contribute to socioeconomic inequality, it should not be interpreted as causal. Second, some important variables deemed to have association with stunting but not measured during 1996 survey were omitted. Third, analysis of dichotomous rather than continuous variables arguably weakens the power of statistical tests [71] but in exchange for easy interpretation and presentation of results we preferred the former. Besides, in case of stunting the cut off points are standardized and recognized world over and the sample sizes are large enough to greatly reduce this concern. In a previous study, conclusions did not change radically when height-for-age z-scores were used as outcome instead of stunting [19]. Fourth, the use of wealth indices for the measurement of socioeconomic position in low income countries is criticized for being sensitive to choice of assets and for not reflecting short term economic shocks [72] and food affordability [31]. However, in absence of direct measurements of household wealth, such asset based index is a good proxy for household socioeconomic status. Fifth, the design of the 1996 and 2016 NDHS may not match completely but since we did not conduct pooled analysis, the estimates from respective surveys stand on their own and proportions should be comparable without much concern.

By applying standard analytical methods our results shed much needed light on the unequal progress in stunting decline in Nepal and has several implications. Nutrition sensitive interventions delivered via a multi-sectoral approach, including increasing overall investment in health and education is key. Increasing coverage of nutrition specific interventions using a life cycle approach in groups that are being left behind may address intergenerational stunting. Although recent efforts for policy coherence across the sectors [22] and rolling out of community level nutrition programmes is commendable; current strategies require targeted approach to address inequalities. By enhancing outreach services and strengthening the community system of service delivery with a focus on poor, disadvantaged and marginalized groups, coverage could be improved across all segments of the population. Efforts for poverty alleviation should go hand in hand with direct investments for reduction of undernutrition primarily in the poorest segments of the population. It is necessary to tackle structural factors that cause unequal wealth distribution through social protection programmes. They can also serve as a delivery platform for nutrition-specific interventions, potentially increasing their scale, coverage and effectiveness.

With the current average annual rate of reduction Nepal will not achieve the World Health Assembly’s target to reduce stunting to 24.3% by 2025 [22] which will in turn offset its course towards SDG 2030 target as well. Interventions should be targeted at the subnational level where the factors causing inequalities are rife. The mountain zone [73], Karnali province and Province 2 [74] not only have relatively higher rates of poverty but also lower levels of education attainment among women and higher total fertility rate [10]. Within these regions, rural areas and communities that suffer higher levels of deprivation should be prioritized to reduce inequalities in stunting. In doing so, leadership of the local governments will be critical in the new federal governance structure. The focus should be on community based primary care approaches using community health workers and volunteers who in the past have successfully contributed to reducing equity gaps and improving access [75].

## Conclusions

Despite remarkable improvements in average stunting over the past two decades, substantial socioeconomic inequalities in stunting exists in Nepal. Not only health system functions such as controlling fertility and improving maternal health but also factors beyond the scope of health authorities and care delivery system such as asset-based wealth distribution and education are at play in determining inequalities. In the past two decades, the better off segments of the population have experienced larger and faster decline in stunting compared to the least affluent and contribution of household wealth to inequalities has increased. Policy interventions that are tailored to inequality patterns, which reach the most disadvantaged and vulnerable groups, might help to change these trends. Multi-sectoral efforts are needed to target nutrition specific and nutrition sensitive programs including social protection for the poor. Political commitments need to be translated in to large scale programs. Nevertheless, for targeting priority populations, subnational information might be needed, which is an area for future studies to delve into.

## Abbreviations

ANC: Antenatal Care
BMI: Body Mass Index
C: Concentration Index
NDHS: Nepal Demographic and Health Survey
NFHS: Nepal Family Health Survey
SD: Standard Deviation
SDG: Sustainable Development Goals
WASH: Water, Sanitation and Hygiene
WHO: World Health Organization

## Footnotes

1 Topographically, Nepal is divided in to three distinct ecological belts along a south-to-north transect: *Terai* (or plains) constitutes 23%, Hill constitutes 42% and Mountain constitutes 35% of total land. According to the latest national population census [57], 50% of the population live in the *terai*, about 42% live in the hills and only about 7 % live in the mountains. Because of the harsh terrain, transportation and communication facilities are very limited in the mountain zone.

2 The new constitution of Nepal which came into effect on September 2015, restructured the country in to a federal democratic republic governed with three levels of government: a federal level, seven provinces and 753 local governments. The seven provinces are (starting from the East): Province 1, Province 2, Province 3, Gandaki Province, Province 5, Karnali Province and Sudur Paschim Province. Province 1, 2, 3 and 5 are yet to be named.

3 Caste/Ethnicity groups as recoded by NDHS: Hill *Brahmin*, Hill *Chhetri*, *Terai Brahmin/Chhetri*, Other *Terai* caste, Hill *Dalit*, *Terai Dalit*, *Newar*, Hill *Janajati*, *Terai Janajati*, Muslim, Other.
